# Self-perceived problems of Afghan asylum seekers and refugees and their experiences with a short psychological intervention

**DOI:** 10.1186/s12889-023-17076-7

**Published:** 2023-11-03

**Authors:** Viktoria Kantor, Dina Weindl, Jennifer Schiess-Jokanovic, Lucia Verginer, Brigitte Lueger-Schuster, Matthias Knefel

**Affiliations:** Department of Clinical and Health Psychology, Faculty of Psychology, University of Vienna, Wächtergasse 1, Vienna, 1010 Austria

**Keywords:** Afghan refugees, aPM+, Transdiagnostic shot intervention, PSYCHLOPS, Subjective symptom burden, Post migration living difficulties

## Abstract

**Background:**

The present study examined Afghan asylum seekers’ and refugees’ self-perceived problems, and their experiences of treatment with an adapted version of the brief transdiagnostic psychological intervention “Problem Management Plus” (aPM+). Specifically, the study explored which problems trouble them most and how these problems influence their daily functioning. Further, it examined how various standardized outcome measures correlate with these subjectively perceived problems.

**Method:**

This study is part of a larger RCT study (PIAAS study) in which 88 Afghan asylum seekers and refugees were randomly allocated either to aPM + in addition to treatment as usual (aPM+/TAU) or TAU alone. The presented study uses a multi-method approach consisting of two parts: First, we investigated participants’ self-identified problems and subjective functional impairment using quantitative and qualitative assessment in both the aPM+/TAU and TAU group (n = 88). Second, we conducted in-depth qualitative interviews with a subsample of the aPM+/TAU group (n = 24) to gain a deeper understanding of participants’ personal experiences with aPM + and to obtain suggestions for improvement. Spearman correlations were applied for quantitative data, and deductive and inductive approaches of thematic analysis were used for qualitative data.

**Results:**

We identified six main themes of self-perceived problems (primary post-migration living difficulties, general mental health problems, interpersonal stressors, secondary post-migration living difficulties, mental health problems specifically associated with stress, and somatic problems) and their consequences, as well as subjective functional impairment. Standardized measures of general mental health, posttraumatic psychopathology, and quality of life did not correlate with the intensity of self-perceived problems. aPM + was mostly perceived positively, and few participants had recommendations for its improvement.

**Conclusion:**

The study aimed at giving a voice to Afghan trauma survivors to inform service providers and policy makers about their needs. Based on their expertise, future interventions can be tailored to their actual needs and optimized in terms of practical use. aPM + proved to be a positively perceived intervention that reduces subjective symptom burden and facilitates daily functioning. Culture-sensitive treatments within (mental) health services should increase service utilization and improve (mental) health in the long term.

**Supplementary Information:**

The online version contains supplementary material available at 10.1186/s12889-023-17076-7.

## Background

Afghans represent one of the largest populations of refugees and asylum seekers worldwide. More than 8.2 million Afghan refugees^1^ live in Central Asia and the Islamic Republics of Iran and Pakistan [1]. In Europe, Afghans represented the second largest group of recent arrivals in the year 2021 and beginning of 2022 [2], and constituted the largest group of refugees in Austria in 2022 [3]. Refugees and asylum seekers have often grown up in a politically unsettled and insecure environment and have frequently faced violence, repression, and discrimination. Indeed, Afghan refugees^1^ living in Austria and elsewhere are particularly vulnerable, as compared to refugees from other origins. On average, those from Afghanistan have a lower educational level and may have different mental health literacy approaches to the host country, which predicts poorer mental health [4, 5]. Moreover, they show higher rates of posttraumatic stress disorder (PTSD) and anxiety/depression [6], have to wait longer for positive asylum decisions [7], and have lower chances of obtaining asylum [8]. Considering these obstacles, it is important to gain more knowledge about the subjective perspective of Afghan refugees. To offer helpful psychosocial support, mental health professionals need to understand their problems and their perceptions of psychological interventions.

Previous reviews have reported varying prevalence rates of mental health disorders in refugees [9–11] but have consistently demonstrated a substantially increased risk of experiencing mental health disorders [12, 13] compared to the general population. Experiences of hardship and traumatic events in the home country, during flight, and in the host country (post-migration living difficulties; PMLD) contribute to symptoms of distress [14] and functional impairment [15]. PMLD and their impact on an individual’s stress levels differ according to the given circumstances in the specific host country [16]. Furthermore, high-income countries’ concepts of mental health disorders, their assessment, and treatment interventions may fail to cover all aspects of distress and individuals’ needs in cross-cultural settings [17]. Research has shown that barriers to accessing mental health care across the European Union are high, with a lack of understanding of the treatment needs of refugee populations leading to low mental health service use [18]. Thus, it is important not only to include predefined measurements and constructs but also to follow a person-centered research and treatment approach considering subjective perspectives, cultural background, and social circumstances [19].

While studies have demonstrated the effectiveness of evidence-based treatments for PTSD in refugee populations [20–23], research on psychosocial interventions addressing other disorders (such as anxiety, somatization, or depression) are sparse [11]. Transdiagnostic approaches target common elements across disorders and address multiple disorders simultaneously [24], and thus represent appropriate low-intensity treatments. Such approaches have been associated with positive treatment outcomes such as symptom improvement, reduced comorbidities, improved social functioning, client satisfaction and therapeutic alliance, and positive treatment expectations [25–27]. Nevertheless, previous studies that considered transdiagnostic approaches showed methodological weaknesses and examined small sample sizes [24], and in particular, evidence in refugee and asylum-seeking populations is lacking [28]. Problem Management Plus (PM+) is a short-term transdiagnostic intervention that was developed to address mental health problems in people exposed to adversity [29, 30]. It was originally designed as a low-threshold treatment offered by lay health workers, which is of high relevance in refugee populations as it facilitates psychological interventions for a larger number of help-seeking refugees within a short time period. Initial analyses of the intervention have revealed good effectiveness in terms of reducing depression, anxiety, PTSD symptoms, functional impairment, and self-identified problems, and it has been shown to be feasible in different populations [31–34]. Perera et al. [35] found that the intervention was associated with improved subjective well-being, a reduction in self-identified problems, and better quality of life in Venezuelan refugees and migrants. It has further shown good acceptance by Syrian refugees [34].

The current study is part of the PIAAS project (a brief transdiagnostic psychological intervention for Afghan asylum seekers and refugees in Austria), which aimed to investigate the effectiveness of an adapted version of PM+ (aPM+) for Afghan refugees living in Austria [36]. To consider participants’ PMLD and cultural background, we followed a person-centered qualitative research approach. First, we sought to gain a better understanding of the subjective burden of treatment-seeking Afghan refugees. To this aim, we set out to identify participants’ self-perceived problems and their consequences, as well as their subjective functional impairment. Second, using standardized questionnaires, we aimed to investigate the associations of participants’ general mental health, posttraumatic psychopathology, and quality of life with their self-reported burden in order to understand their treatment needs. Third, we wished to gain greater insight into participants’ subjective perceptions of aPM + in order to facilitate and promote a low-threshold person-tailored treatment approach for this specific population in the future. The current study is the first of its kind with Afghan refugees in Vienna, Austria. Given that Afghans in Austria represent a group of individuals that show a higher vulnerability to mental health disorders than the general population, it is crucial to achieve an in-depth understanding of their experiences and to establish suitable treatment interventions.

## Method

The PIAAS project aimed to investigate self-perceived problems and correlations of participants’ general mental health, complex posttraumatic stress disorder (c-PTSD), and quality of life with self-reported problem severity. Furthermore, a subsample of the main PIAAS project was asked to discuss their personal experiences with aPM + and provide suggestions for improvement. Within the scope of the project, an extra session was developed to practice strategies to cope with PMLD. The main study was designed as a prospective, single-center, assessor-masked, individually randomized, two-group superiority trial.

The current study uses quantitative and qualitative data from the group receiving aPM + in addition to treatment as usual (aPM+/TAU) and the TAU group from the baseline (t0) and follow-up assessment (t1) as well as qualitative data from in-depth interviews. The study consists of two parts: First, we investigated participants’ self-identified problems and subjective functional impairment by conducting quantitative and qualitative assessments with the treatment and control group (n = 88) of the PIAAS project. Second, we conducted qualitative in-depth interviews with a subsample of the treatment group (n = 24) to investigate participants’ opinions about aPM+.

If participants mentioned during the interviews or treatment sessions that they had been subjected to domestic violence, they were provided with information about Austrian “protection against violence” laws [37] and contact numbers for counseling and help-giving facilities in Vienna. The authors assert that all procedures contributing to this work comply with the ethical standards of the relevant national and institutional committees on human experimentation and with the Helsinki Declaration of 1975, as revised in 2013.

### Setting and data collection

The assessments, intervention, and in-depth interviews were mainly conducted in the Outpatient Unit Research, Teaching, and Practice of the Faculty of Psychology at the University of Vienna. Data were collected face-to-face, with trained clinical psychologists and Dari-speaking interpreters, in a confidential room to ensure privacy. Reading material was translated into Dari. The self-report measures, which were administered as a structured interview due to a high rate of illiteracy in the sample, were implemented one week before the first session of the intervention and one week after the final session. The psychologists carrying out the assessments were blinded to the group allocation at both time points. The in-depth interviews took place between 5.0 and 80.4 (MD = 52.9, IQR = 35.4) weeks after the final intervention session. All in-depth interviews were audio-recorded, transcribed using the transcription software f4transcript (Edu-version), and anonymized. Finally, an independent research assistant compared all transcripts with the audio recordings. Audio files were permanently deleted following data analysis. Demographic information was collected within the main study [36]. Since the data collection took place during the SARS-CoV2 pandemic, some interviews had to be carried out by telephone. For the face-to-face interviews, we followed the Austrian governmental measures to contain the pandemic that were in place at the time (face masks, 2-meter distance, sneeze screen, frequent ventilation).

#### Problem Management Plus, adapted (aPM+)

aPM + comprises six individual sessions that are delivered once a week and involve different cognitive and behavioral strategies. It aims to empower traumatized asylum seekers and refugees to cope with emotional (e.g. stress, mood disorders, sleeping difficulties) and practical problems (e.g. organizing help, structuring daily routines). The original PM + manual comprises four core therapeutic strategies: “Managing Stress”, “Managing Problems”, “Get Going, Keep Doing”, and “Strengthening Social Support” [30]. In aPM+, participants can further address PMLD in an extra session specifically developed for this purpose. They can choose whether they would prefer to focus on strengthening anger regulation or on enhancing self-efficacy, depending on their subjective needs. Strengthening anger regulation includes psychoeducation and several regulation strategies. To enhance self-efficacy, participants draw a so-called “tree of capabilities”, an intervention that aims to reconnect them with their personal strengths, achievements, and empowering resources [36].

### Instruments and in-depth interviews

Several instruments were used for the structured face-to-face interviews with participants of the treatment and control group (n = 88). All instruments showed good reliability in terms of internal consistency in the current sample [for details, 36]. The person-centered brief questionnaire Psychological Outcome Profiles [PSYCHLOPS; 38] investigates participants’ most troubling problems and their subjectively perceived consequences of these problems. The scale comprises three free-text items rated on a 6-point severity scale (from “0 = not at all” to “5 = severely affected”) and an item covering the duration of the problem (from “under one month” to “over five years”). Additionally, one item asks about general well-being during the last week, rated on a 6-point scale (from “0 = very good” to “5 = very bad”). The four ordinally scaled items can be summed to form an overall severity score ranging from 0 to 20. Although PSYCHLOPS is a self-report questionnaire, we administered it within the overall structured interview for reasons of comprehensibility. The General Health Questionnaire 28 [GHQ-28; 39] assesses somatic symptoms, anxiety, insomnia, social dysfunction, and severe depression. It has good psychometric properties and has been frequently used in different cultural populations [40]. We used the existing German and Dari versions of the scale, and internal reliability at baseline was acceptable (Cronbach’s α = 0.75). The International Trauma Questionnaire [ITQ; 41] assesses ICD-11 PTSD and complex PTSD with 18 self-report items. We used the existing Dari version of the ITQ, and the internal reliability at baseline was good (Cronbach’s α = 0.81). The WHO Quality of Life Questionnaire [WHOQOL-BREF; 42] contains 26 items rated on a 5-point Likert scale, covering four domains: physical health, psychological well-being, social relationships, and environment. We used the existing Dari/Farsi version, and the reliability of the WHOQOL at baseline was good (Cronbach’s α = 0.81).

To investigate participants’ experiences with aPM+, a semi-structured interview guide was developed [43, 44]. It contains a face sheet on which the interviewer can write down the date and duration of the interview as well as special remarks (e.g. telephone interview; “participant felt tired during the interview”), and open-ended interview questions. The interview began with an introduction and continued with a number of open-ended as well as follow-up questions regarding whether/how aPM+ (1) was helpful for the participants and (2) influenced their everyday life, (3) whether specific strategies were helpful, and (4) what was problematic. The interview guide ended with questions regarding the improvement of the intervention based on participants’ personal experiences. A copy of the interview guide (in German) is available from the corresponding author upon reasonable request.

### Participants

All 88 participants were adult Dari-speaking Afghan refugees living in Austria (Table 1). They were recruited through waiting lists of Austrian non-governmental organizations (NGOs) that provide psychological and psychiatric help for treatment-seeking refugees and asylum seekers (inclusion criterion a). NGO staff members informed potential participants about the study and forwarded contact details to the research team if they were interested. The research team contacted and informed all potential participants and arranged appointments for the initial assessment. To participate in the study, individuals needed to show elevated psychological distress (inclusion criterion b) based on a screening questionnaire (RHS15). We excluded individuals who presented with a condition requiring other treatment (acute suicidality, severe mental disorder such as psychotic disorders or substance dependence), individuals who showed severe cognitive impairment based on definitions provided in the Mental Health Gap Action Programme (mhGAP) Intervention Guide [45], and individuals who were currently receiving trauma-focused treatment. These exclusion criteria were assessed by the clinical psychologist during the baseline assessment. An independent researcher who was not involved in the study randomized participants into aPM+/TAU or TAU alone. Post-assessments were scheduled one week after the sixth intervention session, or seven weeks after baseline assessment for the control group. Thus, all participants were invited to two assessment time points prior to the qualitative interview.

All research steps were planned and implemented in accordance with the Consolidated Criteria for Reporting Qualitative Studies [COREQ; 46].

The institutional review board of the University of Vienna (reference numbers: 00356 and 00445) approved the study. Participants provided informed consent. If they did not wish to answer individual questions, they could decline to answer without providing any reason.

**Table 1:** Sample characteristics

	PSYCHLOPS sample			aPM + sample
	Total sample (n = 88)	TAU (n = 39)	aPM+/TAU (n = 49)	aPM+/TAU (n = 24)
Gender^a^ (female) n (%)	39 (44.3)	20 (51.3)	19 (38.8)	12 (50.0)
Age M (SD)	34.3 (13.8)	37 (14.4)	32.2 (13.0)	35 (13.6)
Marital Status n (%)				
Single	36 (40.9)	13 (33.3)	23 (46.9)	8 (33.3.))
Married	44 (50)	23 (59)	21 (42.9)	12 (50.0)
In a relationship	2 (2.3)	1 (2.6)	1 (2.0)	1 (4.2)
Divorced	4 (4.5)	1 (2.6)	3 (6.1)	2(8.3)
Widowed	2 (2.3)	1 (2.6)	1 (2.0)	1 (4.2
Asylum status n (%)				
Secure asylum status^b^	39 (44.3)	15 (38.5)	24 (49.0)	12 (50.0)
Insecure asylum status	49 (55.7)	24 (61.5)	25 (51.0)	12 (50.0)

^a^The term gender refers to participants’ self-identified gender

^b^Secure asylum status is defined as having a long-term perspective in Austria, i.e. unconditional permit of residency for more than a year and free access to the labor market

### Data analysis

#### Data analysis of PSYCHLOPS

Initially, the coders started with a deductive approach following Braun & Clarke (2006) [47], based on categories developed by Robinson et al. (2006) [48]. We assumed that similar answers would emerge, and thus planned to assign them to existing categories for the domain “problem” (interpersonal, past event, state of mind, somatic, self-evaluation, competence/performance, material issues) and for the domain “consequences of the problem” (competence/performance, interpersonal, frame of mind, resolution and progression, self-evaluation, somatic). However, upon reviewing the participants’ answers, it became clear that the described problems were different from those of other samples, e.g. talking therapy patients [48] and primary care patients, and therefore did not match the pre-existing themes. Hence, the data analysis was adapted into an inductive approach. First, two independent coders (VK, DW) transferred every free-text response of the three subject areas (problem 1, problem 2, functional consequences) into a code that summarized the content of the response. The coders compared and discussed all codes until both agreed on the final wording of the codes. Next, VK and DW independently grouped all codes into distinct subthemes, compared their results, and resolved disagreements through discussion. They then introduced the final coding scheme, including all codes and subthemes, to two other researchers (MK, JSJ), who independently assigned the codes to the subthemes. MK and JSJ compared their assignments to the assignments of VK and DW, and calculated a good interrater reliability (Cohen’s kappa = 0.94). Three subthemes were reworded following discussion. Finally, VK and DW identified patterns among these subthemes and generated broader themes. In the last step, MK and JSJ reviewed these themes by comparing them with the original free-text responses. Any disagreement was resolved by discussion. All themes and subthemes were finally translated from German into English.

#### Data analysis of PSYCHLOPS severity score and further outcome measures

To quantify the association between the overall severity of self-perceived problems and participants’ general mental health, posttraumatic psychopathology, and quality of life, we calculated the correlations between the scores on the respective questionnaires. As the data of the PSYCHLOPS were not normally distributed, we used Spearman correlation (Shapiro-Wilk test *p* < 0.01).

#### Data analysis of the in-depth interviews regarding participants’ experiences with aPM+

Since the analysis addressed specific research questions, a deductive approach of thematic analysis [47] was applied. After transcription, the interviewers compared all transcripts with the audio files to double-check for consistency, and corrected them if necessary. All transcripts were transferred to the software program MAXQDA 2020 [49]. Two researchers (DW, VK) read all transcripts in order to familiarize themselves with the content and developed the analysis strategy. In an initial step, they independently highlighted five randomly chosen interview transcripts regarding all relevant quotes in order to answer the three main questions: (1) Which parts of aPM + did participants experience as helpful? (2) How did aPM + influence participants’ subjective well-being? (3) How would participants improve aPM+? Since inter-rater agreement was very high (Cohen’s kappa = 0.94), DW and VK continued highlighting relevant quotes independently, each covering one half of the remaining interviews. In the case of uncertainty during the process, the researchers consulted each other to clarify questions. The resulting data set included all relevant quotes divided into three main sections based on the main questions. DW and VK independently coded the first third of the quotes of every section, and then compared and modified them. Next, VK coded all quotes that were assigned to the first research question and DW coded all quotes that were assigned to the second and third research questions. New codes were constantly reviewed, discussed, and modified, and existing codes were sometimes modified based on discussion. DW and VK examined all codes and collated those that fit together into initial themes. Finally, all resulting themes were reviewed and checked in relation to codes and associated quotes, defined and named. During all steps of the analysis, disagreement was resolved either by discussion or by consulting two other authors (MK, BLS).

## Results

### Results of part 1 (PSYCHLOPS)

#### Most troubling problems

We identified 26 subthemes encompassing 175 responses regarding participants’ most and second-most troubling problems. These subthemes were merged into six main themes, which were ordered according to number of respondents: *primary PMLD, general mental health problems, interpersonal stressors, secondary PMLD*, *mental health problems specifically associated with stress*, and *somatic problems*. Table 2 presents all themes, subthemes, and examples of the original responses.

**Table 2:** Themes and subthemes of participants’ most troubling problems (identified with PSYCHLOPS)

#	Theme^a^	Subtheme	Example	Problem 1 (n)		Problem 2 (n)
[1]	Primary postmigration stressors	-Status of residence -Difficult situation in home country -Separation from family/friends -Language problems	Negative decision in lengthy asylum procedure (m, 21) Burdened by the situation in Afghanistan (m, 63) Concerns about family in Afghanistan (m, 26) Difficulties learning German (f, 21)	8 2 9 4 **23**		16 2 7 5 **30**
				**Total = 53**		
[2]	General mental problems	-Cognitive problems -Mood problems -Stress -Sleep problems -Psychiatric disorder	Suffering from forgetfulness (f^b^, 37) Feelings of loneliness (m^c^, 34) Suffering from stress (m, 20) Sleep disturbances (f, 32) Obsessive compulsive disorder (m, 21)	4 5 3 1 1 **14**		4 14 2 1 0 **21**
				**Total = 35**		
[3]	Interpersonal stressors	-Current exposure to violence -Interpersonal concerns: family -Interpersonal concerns: partner -Interpersonal concerns: others -Interpersonal problems: family -Interpersonal problems: partner -Interpersonal problems: others	Domestic violence (f, 35) Concerns about injured brother (m, 22) Worries about disabled husband (f, 60) Concerns about imprisoned friend (m, 22) Mother-in-law often interferes (f, 24) Relationship problems with husband (f, 54) Conflicts with group leader (m, 26)	3 0 6 1 3 0 2 1 **16**		1 1 5 0 1 6 3 2 **18**
				**Total = 34**		
[4]	Secondary postmigration stressors	-Occupation -Finances -Living situation	Not knowing what to do with oneself, because of work ban (m, 21) Needs money; Payment from social welfare office is delayed (f, 36) Deposit charges are too high on housing market (f, 35)	3 8 4 **15**	3 2 6 **11**	
				**Total = 26**		
[5]	Mental problems specifically associated with stress	-Loss -Stressful memories -Nightmares -PTSD symptoms	Ruminating thoughts about son’s death (f, 55) Stressful memories about past (m, 28) Nightmares about stressful past events (m, 53) Heavily burdened by PTSD symptoms (f, 19)	4 4 3 1 **12**		1 1 1 0 **3**
[6]	Somatic problems	-Physical pain -Health problems	Headache (m, 59) Breast cancer and current hormone treatment (f, 35)	5 2 **7**		2 2 **4**
				**Total = 11**		

*Note*: Problem 1: N = 88, Problem 2: N = 87

^a^Themes are presented in order of most frequent responses

^b^female

^c^male

**Post-migration living difficulties**. When asked about their most troubling problem, the largest proportion of participants (43.2%) mentioned PMLD. Guided by the subthemes’ content, we distinguished between two types of PMLD: *primary* and *secondary PMLD*. Primary PMLD include problems that are specifically present after forced migration, such as being separated from family and friends, struggling with an uncertain future due to troubles with residency status, being negatively affected by the difficult situation in one’s home country, and experiencing problems with the foreign language. Secondary PMLD are problems that can potentially affect anybody but may pose a greater burden for refugees and asylum seekers, such as keeping oneself occupied (e.g. not knowing how to occupy oneself because of unemployment due to a precarious residency status), finances (e.g. being dependent on financial support), and living situation (e.g. living in cramped housing conditions).

**Mental health problems**. We distinguished between two types of mental health problems. The first type referred to those *specifically associated with stress* in line with the ICD-11 category “Disorders Specifically Associated with Stress” (World Health Organization, 2019). Associated subthemes encompassed various reactions to stressful or traumatic events, such as the experiencing loss (e.g. losing a family member; death of a child), ruminating on stressful memories (e.g. stressful memories of the past; stressful experiences), suffering from nightmares, or other PTSD symptoms. Second, all mental states that were not clearly related to traumatic stressors were subsumed under *general mental health problems (GMHP)*: cognitive problems (e.g. concentration problems; forgetfulness), mood problems (e.g. loneliness; sadness), stress (e.g. being under mental/physical pressure; feeling “stressed out”), sleep problems (e.g. waking up frequently; burdened by sleep disturbances), and psychiatric disorders (e.g. OCD; depressive symptoms). *GMHP* were the second most common responses throughout the whole dataset (Table 2).

**Interpersonal stressors**. We identified two different types of *interpersonal stressors*: *Interpersonal concerns* refer to being worried about the well-being of a family member, partner, or friend (e.g. worries about the future of disabled child), while *interpersonal problems* relate to conflicts and difficulties between individuals, and result from interactions (e.g. repeated verbal disputes with husband). Some participants mentioned that they were currently experiencing violence. Although *current exposure to violence* would fit the description of interpersonal problems, we decided to handle this as a special case and thus depicted it separately. All participants who mentioned during the interviews that they were currently experiencing violence received information about the Austrian “protection against violence” laws [37] and contact numbers of counselling and help-giving facilities in Vienna. *Interpersonal stressors* were the third most common responses throughout the dataset.

**Somatic problems**. Compared to other themes, *somatic problems* were rarely mentioned as participants’ most troubling problems (8.0%). We grouped these responses in two subthemes: *physical pain* (e.g. headache; back pain) and *health problems* (e.g. physical health-related problems such as diabetes or cancer).

### Functional impairment

The majority (n = 82, 93.2%) of the 88 participants responded to the question about functionality. The analysis of the dataset resulted in five main (ability to learn and to concentrate in school, ability to engage in activities of daily living, mental health problems, occupation-related ability or skills, and interpersonal stressors) and 17 subordinate themes (Table 3).

**Table 3:** Themes and subthemes of participants’ subjectively perceived functional impairment (identified with PSYCHLOPS)

#	Theme^a^	Subtheme	Example from the original answers	n
[1]	Interpersonal stressors	-Interpersonal problems: others -Interpersonal concerns: family	Concerns about sister who suffers from depression (f^b^, 35) Ongoing conflicts with roommate (m^c^, 18)	1 1
				**2**
[2]	Impairment in activities of daily living	-Listlessness -Housekeeping -Childcare -Dependent on others	Feeling listless to do anything (m, 59) Reduced ability to keep the house tidy and to cook (m, 22) Has the feeling it influences her abilities to take care for her children (f, 35) Needs somebody who accompanies her to appointments (f, 61)	13 11 2 1
				**27**
[3]	Language and learning related problems	-Learning German -Learning -Communicating in German	Influences ability to study German (f, 55) Problems to study for tests in school (m, 23) Cannot talk to neighbors and others (f, 21)	8 18 2
				**28**
[4]	Mental problems	-Mood problems -Cognitive problems -Stress -Sleep problems -Withdrawal from social situations	Cannot feel happy; loss of (m, 23) Forgetfulness (f, 31) Feeling stressed out when doing things (f, 36) Problems to fall asleep (m, 21) Does not want to meet friends; wish to withdraw from others (m, 22)	4 2 4 3 5
				**18**
[5]	Occupation	-Influence on ability to work -Boredom -Work-related skills	Lost job because of the problems (m, 39) Feels constantly bored, because she is not allowed to work (f, 35) Does not know how to manage her time better (f, 21)	4 1 2
				**7**

*Note*: N = 82

^a^Themes are presented in alphabetical order

^b^female

^c^male

About two thirds of the participants (67.1%) described that their problems influenced their ability to learn (especially to learn German) and to concentrate in school, or to engage in activities of daily living. The responses of 18 (21.9%) participants were merged into the theme *mental health problems.* These participants mainly mentioned that their problems influenced their mood, cognitive skills, or sleep, or led to feelings of stress or social withdrawal. Seven (8.5%) participants felt that their problems influenced occupation-related abilities or skills, and two (2.3%) related their problems to interpersonal stressors.

### Quantitative analysis of the PSYCHLOPS

The overall severity score on the PSYCHLOPS was not significantly associated with general mental health (GHQ: r_S_ = 0.04, p > 0.05), posttraumatic psychopathology (ITQ: r_S_ = -0.01, p > 0.05), or quality of life (WHOQOL: r_S_ = 0.13, p > 0.05).

### Results of part 2 (in-depth interviews)

For an overview of the results of part 2, see Table 4.

**Table 4:** Overview of all Themes and most often mentioned subordinated codes per topic

**1. Experiences with aPM+**					
Specific aPM + strategies and interventions	Trust and therapeutic relationship	Generally helpful	Further psychological interventions	Structure	Learning and practicing
Slow breathing (22) Social support (12) Inactivity cycle (8) Tree of resources (7) Physical exercises accompanying slow breathing (5) Problem management (4)	Positive relationship with PM + trainer (17) Talking openly about problems (7) PM + trainer showed empathy (2)	Generally helpful (7) All strategies were helpful (4) Generally good experiences (4)	Positive affirmations (5) Drinking a glass of water (3) Supportive words (2)	Handouts (3) Weekly appointments (2) Reminder per SMS (2)	Learning something new (3)
**2. Reported effects of aPM+**					
Mental	Knowledge	Interpersonal	Behavioral	Physical	Insufficient effect
Feeling better (10) Feeling empowered (5) Being calmer/steadier (5) Being more positive (4) Ruminating less (4)	Regulating oneself better (11) Taking time to solve problems (1)	Engaging more with people (5) Coping better with conflicts/being more patient (5) Seeking social support (2)	Establishing daily structure/activation (5) Being more autonomous (2)	Stomach smaller (1) Sleeping better (1) Having less headache (1)	Cannot recognize impact (5) Ruminating more (1)

#### Experience with aPM+

When asked about their overall experience with aPM+, nearly all participants described their participation as positive and mentioned specific strategies they found particularly useful (Supplementary Table S1). In particular, *slow breathing* (“Managing Stress”) was positively acknowledged: “The most important strategy for me was the breathing exercise. I had a lot of stress/ I couldn’t bear it. Then, I went somewhere alone, I sat down and I started to breathe slowly. That was really helpful” (P18, m, 25). Some participants did not directly mention the breathing exercise but referred to physical exercises to relax the body (e.g. relaxing muscles, shaking arms and legs, slowly moving the head). Others did not mention specific strategies but described aPM + as *generally helpful*.

Another positively mentioned strategy was strengthening *social support*. Participants learned that isolation and social distance might impede proper coping with current practical or emotional problems. Thus, they were supported to (re-)connect with others:

I have learned something else here! About social support/ About being in contact with others. Before, I always hid from others/ or when my friends called me, I told them ‘I don’t have time’ or just ‘I can’t’. Somehow, I just tried to hide and didn’t want to see anybody or to go out with them. However, since I have been here [Note: participated in aPM+], I’ve learned that I need to have contact with others. And now, if others call me/ or I call them and I visit them/ or they come to me, or we go out together. I have much more contact with others now. (P81, f, 56)

Learning about the *cycle of inactivity* and identifying activities in which participants like to engage was likewise described as helpful:

Of course, if you stay home and sleep/ if you lie in bed/ you don’t get out and if you don’t have contact with others, it makes you sick, yes. That was very helpful/ I learned here that when you sit all day at home/ if you don’t leave the flat/ if you don’t do anything, you are probably getting sick/ So, now I go out/ I go for a walk in the park and things like that/ I do that a lot, yes. (P64, m, 59)

The newly developed sixth session was greatly appreciated by the participants. Those who chose to *strengthen self-efficacy* enjoyed drawing their “tree of capabilities”. Some remembered personal skills and resources they had forgotten about, and some felt stronger after reviewing their achievements. One participant reported that he put his tree over the bed in his room, which helps him to keep a positive attitude. Those that chose the strategy *anger regulation* found it to be helpful in difficult situations.

Some participants mentioned that they use various strategies they have learned in aPM+:

Last time, I used this strategy/how do you call it/ the strategy to calm me down when I am angry, the one that helps me to control my anger. For example, I need to focus on the feeling that is right there and feel my body/ and if my fist is clenched, I need to consciously release it. I also learned that it helps me to leave the situation/ just to go to another place. That helped me a lot, especially in some discussions (laughing)/ and also drinking a glass of cold water and slowly breathing in and out. (P42, f, 37)

Many participants described the *therapeutic relationship* with the aPM + trainer as very positive and trustful, and some appreciated being able to talk openly:

I had the chance to talk with her about everything. Even about stuff I couldn’t talk about with others. I have many friends/ many friends, but I couldn’t talk with them about my personal problems. Here [note: aPM + training], I talked about everything that was in my heart. I took everything out of my heart. (P18, m, 25)

Some participants mentioned that the aPM + trainer suggested something or supported them in ways that were not explicitly described in the original manual but may have been applied intuitively, since all aPM + trainers were experienced clinical psychologists. Therefore, we developed the theme *further psychological interventions*, which comprised remarks referring to such therapeutic acts. For example, some participants described positive affirmations as helpful. Finally, several participants referred to the usefulness of the handouts, which they could take home and therefore helped to keep aPM + in mind.

After attending the final aPM + session, 15 participants indicated that they continued with the *slow breathing* (“Managing Stress”). Only a small number continued to use any of the other strategies (Supplementary Table S4).

Although most of the participants shared positive experiences, some reported challenges during their participation of aPM+. Several described difficulties with *specific aPM + strategies and interventions*, particularly “Strengthening social support” and “Managing problems”. A small number of participants stated that the *transfer to everyday* life was difficult. One participant experienced aPM + as helpful during the training but stated that “then, new problems occurred and I stopped doing anything/ I just stopped.” (P36, m, 23).

Some participants had *different expectations* of aPM+. For example, one person hoped to get specific help for dealing with anxiety:

P: Nothing helped against my anxiety/ I suffer from anxiety/ I was afraid, but it [note: aPM + training] was not helpful.

I: It didn’t help you to deal better with your anxiety?

P: No. (P86, m, 26)

In view of the special situation in which participants were living, some saw themselves as faced with *unsolvable problems* and therefore could not find support in the training. Three participants stated that *too many questions (about the past*) were asked: “I didn’t find a solution. Only questions and no answers. It was boring for me to get so many questions and to speak only about one theme every week.” (P14, m, 34) (Supplementary Table S5).

#### Reported effects of aPM+

Participants perceived a positive impact after aPM + training in several domains: *mental, knowledge, interpersonal, behavioral*, and *physical* [50] (Supplementary Table S2). Some participants observed no impact of aPM + at all, which was categorized as a sixth domain, *insufficient effect*. One participant reported that he ruminated more, and “afterwards back at home, I spent the whole night thinking about the past” (P18, m, 25).

Nevertheless, the majority of participants reported positive experiences, particularly in terms of changes on the *mental* level: “Training was good. I joined in and did the movements. Afterwards, I felt better. I had a good feeling later.” (P45, m, 20). Some felt empowered: “I used to think that I am worthless. (…) and that I cannot achieve anything. (…) during the training I realized that if I want to do something, I could do it (…) achieve it.” (P50, f, 21). Participants reported that they became calmer and some stated that they recognized a more positive attitude in themselves: “When I wake up in the morning, I think that I’m happy, that I feel good. I try to think positively. And I wish for something good and I think to myself, in the future everything will be fine again” (P18, m, 25). Others reported less rumination: “I don’t have to think so much” (P38, m, 24).

Furthermore, participants identified increased *knowledge* about self-regulation:

Some situations are very stressful. For example, this month I have an appointment for my driver’s license. (…). It is difficult because I have to learn a lot before the test and before I go to the test on that day, I will do the breathing exercise that I learned in training. (P83, f, 25)

One participant also acknowledged that: “Okay, I started from scratch. Where am I now? And then I noticed that life doesn’t go all that fast. The problem isn’t solved all at once. That is what I have learned.” (P36, m, 23).

Some participants identified positive changes on the *interpersonal* level, noting that they were seeking social support more often and engaging better with others: “I wanted to stay alone. Back then, I avoided my family and other people. But now I like to have better / more contact with my family, with my children, and have more to do with them.” (P97, f, 40) They also noticed positive effects concerning interpersonal conflicts. For instance, one participant stated, “It has already caused changes in how you talk to other people / how you should behave. That was very helpful.” (P64, m, 59).

On the *behavioral* level, participants established a daily structure and perceived more autonomy: “I even went shopping even though I haven’t done this before. But now I’ve been doing this since training / after the training and now. That’s really helpful.” (P19, m, 57).

Only few participants also reported positive *physical* effects like sleeping better or having fewer headaches (Table S2).

#### Recommendation of aPM+ and its strategies

Half of the participants recommended aPM+ to relatives or friends.

I have told my children that I am here for this training. Then I also contacted other people (…) I contacted my friends again. And they said, “What happened? We see you again. We hear you again.“ I told them that I was going to the University of Vienna for this training and they were happy. My children too, my friends too. And there was also a mother and daughter, I gave them the address and I said, “You could go there too, if you want.“ I think the two of them came too. (…) I was really excited, and I was happy about the training and I told everyone about it. (P81, f, 56)

Slow breathing (“Managing Stress”) was recommended most frequently, while other strategies were scarcely mentioned (Supplementary Table S3).

#### Participants’ suggestions for improving aPM+

Although some participants seemed reticent about suggesting improvements, “Maybe you [the interviewer] know better after all / maybe learn something else that is more helpful (…) there is nothing on my mind. You are a therapist; you know better than I do.” (P31, m, 21), some suggestions were given. Several participants thought that *more treatment appointments* would be helpful, “I think when people get more time, for example, for therapy, it’s much better. I also think that it’s important that you get more appointments /treatment faster” (P18, m, 25). Two participants suggested *more hands-on practice* to increase engagement for future participants.

I think it would be better if there were more hands-on practice. (…) Some people don’t want to leave home, their own apartment, and feel inactive. They are always at home. That’s why I think it would be necessary to do more exercises. (…) You had the strategy “problem management” that helps you to get out of a problem when you look closely at it. I think for these people [future aPm + participants] you really need that kind of hands-on practice. That you do it together with them. Then they can get out of such problems. Solve their own problems.” (P81, f, 56).

Furthermore, participants proposed *canceling one of the strategies*, providing *more information* on several topics (e.g. parenting, work, and learning), *additional medication*, *promoting treatment among Afghan men*, and *talking less about the past* (Supplementary Table S6).

## Discussion

The present study investigated Afghan refugees´ subjective perspective of their mental health and problems. Further, it examined their experience of a standardized treatment program aPM+. Regarding our first aim, we identified six main themes about participants’ most and second-most troubling problems: *primary post-migration living difficulties* (*PMLD), general mental health problems (GMHP), interpersonal stressors, secondary PMLD*, *mental health problems specifically associated with stress*, and *somatic problems)*. The majority (n = 82, 93.2%) responded to the question about functionality, and the analysis resulted in five main themes: ability to learn and to concentrate in school, ability to engage in activities of daily living, mental health problems, occupation-related ability or skills, and interpersonal stressors. Regarding our second aim, we found that standardized measures of general mental health, posttraumatic psychopathology, and quality of life do not correlate with the perceived intensity of these self-perceived problems, underlining the importance of personalized treatment outcome measures. Finally, regarding our third aim, the participants mostly experienced the brief psychological transdiagnostic intervention aPM + as positive and had several recommendations for its improvement.

### Self-perceived problems

**Primary PMLD and GMHP**. The participants most frequently reported *primary PMLD* (e.g. being separated from family/friends, insecure residency status, problems with foreign language, being negatively affected by the difficult situation in one’s home country), which underlines the burdens faced by Afghan refugees compared to the general population. As the second-most common problem, participants mentioned *GMHP* such as cognitive, mood, or sleep problems and psychiatric disorders. This is in line with previous studies reporting that PMLD such as family problems, distress about the asylum process, and unemployment are strongly related to mental health problems [51]. Alemi et al. [52] found negative mental health outcomes among Afghan refugees in the US due to fractured family relations after their flight and the sociopolitical situation in their home country [53]. In a systematic review examining post-migration variables affecting mental health outcomes among asylum-seeking and refugee populations in Europe, Gleeson et al. [54] found correlations between family life, family separation, and mental health outcomes. Interestingly, residency status was not an explanatory variable for mental health status but rather served as a marker for other PMLD variables such as discrimination, loneliness, and language problems. Having a secure residency status was associated with increased access to resources, support, and opportunities. However, none of the studies included in the analysis examined self-perceived problems. In the present study, we found that an insecure residency status seemed to add to participants’ subjectively perceived burden, and is therefore important to acknowledge.

Participants mentioned GMHP as subjectively stressful, and the quantitative evaluation of the General Health Questionnaire [GHQ; 36] revealed a high symptom burden. After treatment with aPM+, participants showed significantly lower GHQ scores [36], illustrating the efficacy of the aPM + training. Interestingly, we found no significant correlations between the overall severity code of personal distress (PSYCHLOPS) and the GHQ. Furthermore, the PSYCHLOPS was not significantly correlated with the quantitative measures of posttraumatic psychopathology (ITQ) or quality of life (WHOQOL). Therefore, it appears that symptom-oriented questionnaires assessing psychopathological profiles do not reflect the full range of problems that are perceived by refugees. To pave the way for an individualized treatment and to increase treatment compliance, it is thus necessary to gain better insight into the individual needs of (Afghan) refugees. In particular, it is important to assess subjective distress beyond psychopathological symptoms.

**Somatic problems**. Previous research reported significant correlations of somatization with lack of information about family members and subjectively perceived need for healthcare [13, 55], with PTSD [56], and with PMLD [55]. At the same time, studies have yielded heterogeneous findings, and country-specific differences concerning somatic problems in refugee samples were reported [19]. In our sample, perceived somatic problems were mentioned less often than other domains, and participants rarely reported positive effects regarding physical pain and health problems after aPM + training. As an explanation for this finding, we assume that participants are less likely to mention these problems in an open interview format as compared to when they are specifically asked about somatic problems with closed-ended questionnaire items.

### Subjective functional impairment

When asked about their most troubling problems before training, approximately two thirds of the participants reported functional impairment concerning performance-oriented activities, language acquisition, and activities of daily living. This is in line with previous studies that likewise revealed functional impairment across psychological domains in individuals with PTSD symptomatology [57, 58]. Participants’ burden due to their subjectively perceived problems did not correlate with any of the standardized measures used in our study. This is an important finding, as it highlights the difference between checklist-oriented assessment and Afghan refugees’ self-perceived problems. Studies that solely employ standardized measures to assess participants’ distress may potentially overlook the problems that the participants themselves perceive as the most stressful, and may therefore be associated with low acceptance rates. We thus encourage future researchers to integrate person-oriented measures of distress into their assessments, and to examine functional impairment, for instance using the WHO Disability Assessment Schedule (WHODAS) [59], which assesses an individual’s functioning in six core domains: cognition/communication, mobility, self-care, interpersonal interaction, life activities, and participation in society. Functional impairment substantially adds to individual subjective burden and needs to be considered when investigating refugee populations or individuals with a history of trauma [60, 61].

In line with previous research, two thirds of our sample reported an impaired ability to learn and to concentrate as one of their main subjectively perceived challenges [62]. Supporting our qualitative findings, a previous quantitative analysis using the same sample also revealed associations of CPTSD symptoms with language acquisition and barriers [63]. One explanation for this finding might be that Afghan refugees represent a very vulnerable group that has been exposed to cumulative risk factors in their home country as well as during and after flight [14]. Unfavorable coping or functional impairment may be further fostered by illiteracy and by a very different cultural and social background in the host country, such as in Austria. This may be further compounded by a lack of culture-sensitive programs to learn (a new language). Considering migration models, it seems advisable to facilitate language acquisition early in order to foster health literacy [64] and acculturation strategies. Feelings of impairment in language and learning skills can negatively influence active coping with PMLD, which can further increase trauma-related symptomatology [65].

### Subjective perception of PM+

After the training, participants reported a positive experience with the aPM + training and that they noticed positive changes in several psychological domains (mental, knowledge, interpersonal, behavioral). For instance, they perceived increased positive feelings and improved attitudes (mental domain), and increased expertise regarding self-regulation (knowledge domain). Interpersonal contact became easier and participants felt more competent in structuring their daily life. These findings correspond to previous research, which likewise reported positive perceptions of the intervention and its strategies [50, 66].

Participants perceived *slow breathing* to be the most useful strategy. This is a very simple intervention that can be easily understood and practiced, is included in every training session, and the positive perception of this strategy was supported by the direct physiological feedback it provided (i.e. relaxation). Similar outcomes were reported by van’t Hof et al. (2018). Participants further appreciated the additional strategy of *tree of capabilities*, which was easy to recall where needed, and helped the participants to re-identify their capabilities. Mental health professionals have also identified these two interventions as their most favored ones [36]. Some participants mentioned that they could not manage to transfer the strategies learned to their everyday life. Moreover, participants suggested more sessions and/or refresher sessions and hands-on practice, similar to the findings of van’t Hof et al. (2018). Madsen et al. [67] reported that regular practice and instructions for small exercises were necessary to support transfer to daily life, effectively reduce symptom burden, and increase positive contact to oneself. Therefore, this can be highlighted as particularly important for future treatment designs.

Interestingly, participants rarely reported somatic symptoms. Research on somatic symptoms in refugee populations has yielded heterogeneous findings [19]. Therefore, to gain a better understanding of the complex interaction between different challenges and somatic symptoms in the present host country of Austria, it would be necessary to investigate the local contextual factors and PMLD affecting the population under study in greater depth. This would further inform the development of an individualized treatment approach that targets those symptoms that cause the greatest distress and increases daily functioning [68].

Furthermore, participants perceived the therapeutic relationship as helpful. This is in line with previous research that found a positive therapeutic relationship to be a reliable predictor of positive treatment outcomes [69] and that cultural sensitivity and awareness of the therapist is associated with improved life satisfaction [70]. Few of the participants wished to talk less about their past. As individuals do not necessarily need to talk about their past during the aPM + training, we assume that these participants considered the pre- and post-assessment to be part of the training.

Participants reported that they recommended the training to family and friends because they were able to feel positive effects of the training. Therefore, it is important to note that peers could also serve as facilitators of mental health service use, and future mental health interventions should consider this within their treatment designs [71, 72]. Interestingly, when participants were asked about their recommendations to improve the treatment, they could only think of few, and seemed to be very reluctant to provide feedback. We assume that this might be linked to a general low Western health literacy in the present sample [73] and the subjective perception that “experts know best”.

### Limitations

Several limitations of the present study should be mentioned. First, due to the SARS-CoV2 pandemic and the rigorous restrictions for the Austrian population, the period between the last training session and the qualitative interviews varied between 5.0 and 80.4 (MD = 52.9, IQR = 35.4) weeks. Therefore, it might have been difficult for some of the participants to remember the treatment well and give constructive feedback. Second, also due to COVID-19 restrictions, we were forced to conduct some of the interviews by telephone or video call because in-person meetings were prohibited. This, as well as working with translators, might have caused a certain bias in the interviews. Nevertheless, previous research demonstrated that both in-person and telephone interviews are accurate [74] and that the use of interpreters has a positive impact on mental health service utilization and treatment outcomes [75, 76]. Potential ethical issues arising from this procedure were discussed in the research team, and given that all participants were already known to us in person, we did not identify any conflicting ethical problems. Third, our results may not generalize to Afghan refugees as a whole, as our sample represents a help-seeking group with clinically relevant mental health problems. Fourth, the age distribution was imbalanced (mainly young adults), which may have led to some bias. Fifth, more participants of TAU had an insecure asylum status, and we do not know whether this may have had an impact on their subjective perceptions. Sixth, it is possible that we did not reach individuals who could not find the energy and motivation to apply for treatment. Finally, our study took place in Vienna, Austria, and our results may not generalize to refugee populations living in other cities and countries.

## Conclusion

The results of this study suggest that aPM+, a transdiagnostic psychological intervention, is a feasible and well-accepted intervention for Afghan refugees living in Austria. The study highlights the importance of considering subjectively perceived symptom burden to support treatment compliance and effectiveness. aPM + provides a culture-sensitive and person-oriented approach, is effective, and fits the specific needs of migrants and refugees. In the presented qualitative reports, it became clear that easily accessible interventions as well as repetitions support ongoing practice of certain strategies and the perceived effectiveness of the treatment. The interventions seem to address the problems associated with PMLD and to increase self-regulation. Thus, aPM + appears to be an appropriate intervention to reduce subjective symptom burden and facilitate daily functioning. In future research and treatment targeting refugees, such small but effective interventions should be considered and implemented into treatment routines. Regular practice and instructions from the therapist are necessary to improve treatment outcomes. Furthermore, it would be advisable to investigate the effectiveness of a planned booster session some weeks/months after completion of the training [50, 66]. Overall, it is important to consider a culture-sensitive adaptation of mental health services and appropriate treatment offers for refugees in order to increase mental health service use and literacy and to improve mental health [77], life satisfaction, and well-being in the long term.

### Electronic supplementary material

Below is the link to the electronic supplementary material.

Supplementary Material 1

Supplementary Material 2

Supplementary Material 3

Supplementary Material 4

Supplementary Material 5

Supplementary Material 6

Supplementary Material 7

## Data Availability

The datasets used during the current study are available from the corresponding author on reasonable request.

## Footnotes

1 In this paper, we use “refugees” as an umbrella term to denote all asylum seekers regardless of the recognition of their claim.
