# Supplementary figures and images for: Self-perceived problems of Afghan asylum seekers and refugees and their experiences with a short psychological intervention
Source: BMC Public Health. 2023 Nov 3;23:2157. doi: 10.1186/s12889-023-17076-7 (PMC10625214; doi:10.1186/s12889-023-17076-7)

## Supplemental Figure 1.

### Participant Flow

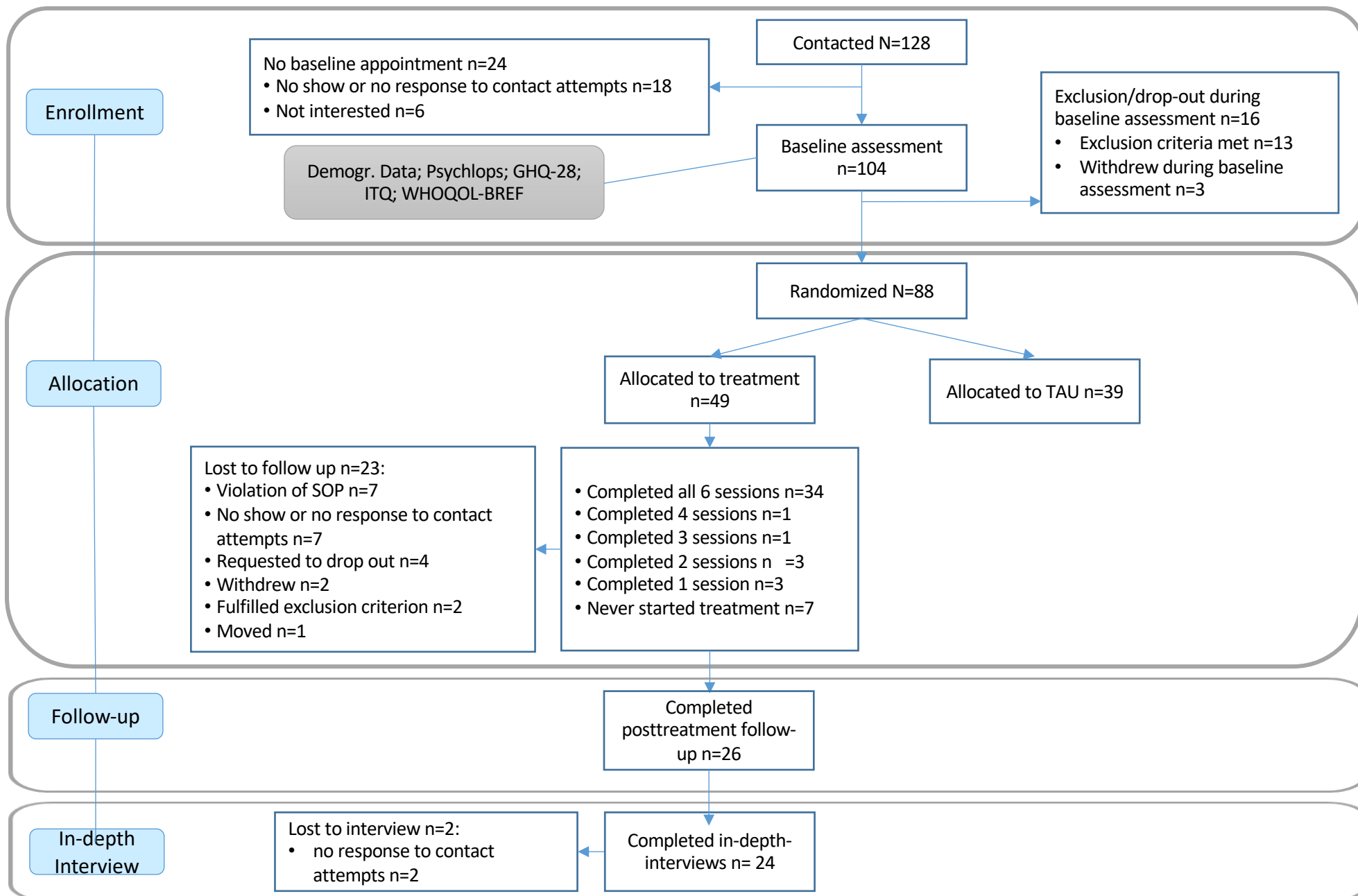

Supplement: Supplementary file 1 — Supplementary Material 1 [file 12889_2023_17076_MOESM1_ESM.pdf]
