# Supplementary material for: Self-perceived problems of Afghan asylum seekers and refugees and their experiences with a short psychological intervention
Source: BMC Public Health. 2023 Nov 3;23:2157. doi: 10.1186/s12889-023-17076-7 (PMC10625214; doi:10.1186/s12889-023-17076-7)
Supplement: Supplementary file 2 — Supplementary Material 2 [file 12889_2023_17076_MOESM2_ESM.docx]

**Table S1.** *Participants’ positive experiences with aPM+*

| Themes | Codes (n^1^) | Participants  P# (gender, age^2^) | Selected quotes (P#, gender, age) |
| --- | --- | --- | --- |
| Specific aPM+ strategies and interventions | Slow breathing (22) | P01 (f, 50)  P05 (f, 54)  P14 (m, 34)  P18 (m, 25)  P19 (m, 57)  P20 (m, 25)  P25 (m, 36)  P31 (m, 21)  P38 (m, 24)  P42 (f, 37)  P45 (m, 20)  P47 (f, 36)  P51 (f, 59)  P56 (f, 32)  P64 (m, 59)  P66 (f, 35)  P81 (f, 56)  P83 (f, 25)  P86 (m, 26)  P90 (m, 22)  P97 (f, 40)  P99 (f, 24) | “The most important strategy for me was the breathing exercise. I had a lot of stress/ I couldn’t bear it. Then, I went somewhere alone, I sat down and I started to breath slowly. And that was really helpful.” (P18, m, 25)  “Breathing in with the belly/ that was also very helpful/ I still do it every day.  I^3^: You are still doing that.  P: If I don’t do it today, I will do it the day after/ but yes, I am still doing it.” (P19, m, 57) |
|  | Social support (12) | P14 (m, 34)  P18 (m, 25)  P19 (m, 57)  P31 (m, 21)  P36 (m, 23)  P47 (f, 36)  P50 (f, 21)  P64 (m, 59)  P66 (f, 35)  P81 (f, 56)  P86 (m, 26)  P97 (f, 40) | “I have learned something else here! About social support/ About being in contact with others. Before, I always hid from others/ or when my friends called me, I told them “I don’t have time” or just “I cannot”. Somehow, I tried to hide and didn’t want to see anybody or to go out with them. However, since I was here [Note: engaged in the PM+ training], I learned that I need to have contact with others. Now, if others are calling me/ or I call them and I visit them/ or they come to me, or we go out together. I have much more contact with others now!” (P81, f, 56) |
|  | Inactivity cycle (8) | P14 (m, 34)  P19 (m, 57)  P38 (m, 24)  P45 (m, 20)  P51 (f, 59)  P64 (m, 59)  P66 (f, 35)  P83 (f, 25) | “Of course, if you stay home and sleep/ if you lie in bed/ you don’t get out and if you don’t have contact with others it makes you sick, yes. That was very helpful/ I learned that here, that when you sit all day home/ if you don’t leave the flat, if you don’t do anything you are getting sick/ So, now I go out/ I go for a walk in the park and things like that/ I do that a lot, yes.” (P64, m, 59) |
|  | Tree of resources (7) | P01 (f, 50)  P36 (m, 23)  P45 (m, 20)  P50 (f, 21)  P51 (f, 29)  P83 (f, 25)  P99 (f, 24) | “I think drawing the tree was really helpful. If you have problems/ if you’re feeling bad, you really get hope/ you start feeling hopeful.” (P36, m, 23) |
|  | Physical exercises accompanying slow breathing (5) | P01 (f, 50)  P38 (m, 24)  P45 (m, 20)  P51 (f, 59)  P66 (f, 35) | “First these exercises/ turning the head from side to side, up and down, back and forth/ moving the shoulders to the front and back/ and the breathing exercise after that.” (P66, f, 35) |
|  | Problem management (4) | P18 (m, 25)  P56 (f, 32)  P97 (f, 40)  P99 (f, 24) | “It was good for me to talk with somebody about my problems. And I found some solutions for my problems/ Now, I can live better with them. That was very good for me.  I: How did the PM+ training help you with that?  P: I learned how to find an easy way to solve everyday problems/ I learned how I could manage these things better. I have to do it in baby steps/ in small steps starting to change things that are troubling me. And I write it down/ that’s how she [note: PM+ trainer] showed it to me.” (P97, f, 40) |
|  | Dealing with feelings of aggression (1) | P42 (f, 37) | “In the last time I used this strategy/how do you call it/ the strategy to calm me down when I am angry, the one that helps me to control my anger. For example, I need to focus on the feeling that is right there and to feel my body/ and if my fist is clenched to consciously release it. I also learned that it helps me to leave the situation/ just to go to another place. That helped me a lot, also in some discussions (laughing)/ and also drinking a glas of cold water and slowly breathing in and out.” (P42, f, 37) |
| Trust and therapeutic relationship | Positive relationship with PM+ trainer (17) | P05 (f, 54)  P18 (m, 25)  P19 (m, 57)  P25 (m, 36)  P31 (m, 21)  P36 (m, 23)  P38 (m, 24)  P42 (f, 37)  P50 (f, 21)  P51 (f, 59)  P56 (f, 32)  P64 (m, 59)  P66 (f, 35)  P81 (f, 56)  P83 (f, 25)  P90 (m, 22)  P99 (f, 24) | “I was feeling very well with the psychologist/ she was good (...)^4^/ And I really trusted her. She told me to do things like this and like that/ she showed me how to do things better. And I trusted her a lot.“ (P51, f, 59) |
|  | Talking openly about problems (7) | P18 (m, 25)  P31 (m, 21)  P36 (m, 23)  P42 (f, 37)  P50 (f, 21)  P64 (m, 59)  P97 (f, 40) | “She [note: PM+ trainer] was very friendly, she was very good. I had the chance to talk with her about everything. Even about stuff, I couldn’t talk about with others. I have many friends/ many friends, but I couldn’t talk with them about my personal problems. Here [note: PM+ training], I talked about everything that was in my heart. I took everything out of my heart.” (P18, m, 25) |
|  | PM+ trainer showed empathy (2) | P18 (m, 25)  P66 (f, 35) | “I: How would you describe the relationship between you and the psychologist [note: PM+ trainer]?  P: I had a good feeling.  I: Can you describe that feeling more detailed?  P: When I was talking, she was feeling it/ feeling with me and she understood the problems I had. She understood my feelings.“ (P66, f, 35) |
|  | Further codes: Being in contact with somebody/PM+ trainer (1), Starting to trust others again (1), Trusting the interpreter (1), Friendly attention (1), First experience with a psychological intervention (1) | | |
| Generally helpful | Generally helpful (7) | P18 (m, 25)  P19 (m, 57)  P38 (m, 24)  P42 (f, 27)  P45 (m, 20)  P51 (f, 59)  P90 (m, 22) | “In the beginning, it was very tough for me. When I experienced something unpleasant, I was thinking about it for many days. That was difficult for me. Now, it is not like that anymore!  I: What has changed?  P: You have helped me. These sessions I attended here/ the training. It was quite helpful.” (P18, m, 25) |
|  | All strategies were helpful (4) | P01 (f, 50)  P36 (m, 23)  P45 (m, 20)  P83 (f, 25) | “They [note: strategies] have all been helpful. Every one of them was helpful in their way/ depending on the different areas, they all helped me in a certain way.” (P1, f, 50) |
|  | Generally good experiences (4) | P25 (m, 36)  P56 (f, 32)  P66 (f, 35)  P83 (f, 25) | “The training was okay, it had a positive influence, and it was good.” (P56, f, 32) |
|  | Further code: Helpful, but cannot say how it helped (1) | | |
| Further psychological interventions | Positive affirmations (5) | P42 (f, 37)  P51 (f, 59)  P66 (f, 35)  P81 (f, 56)  P86 (m, 26) | “At every appointment, she [note: PM+ trainer] has told me: ‘You are a very strong women! You can do it!’ It was helping me a lot! I felt so strong/ it was very helpful.” (P51, f, 59) |
|  | Drinking a glass of water (3) | P14 (m, 34)  P20 (m, 25)  P42 (f, 37) | “It is good to drink a glass of water and to breathe deeply in and out.” (P14, m, 34) |
|  | Supportive words (2) | P51 (f, 59)  P81 (f, 56) | “She [note: PM+ trainer] told me: “you can be more self-dependent”/ “you can do it”/ She made me feel brave/ I felt braver/ It helped me!” (P81, f, 56) |
|  | Further codes: Setting boundaries (1), Cognitive strategy to deal with intrusive memories (1), Taking a break (1), Taking small steps to change (1), Calming oneself down (1) | | |
| Structure | Handouts (3) | P25 (m, 36)  P56 (f, 32)  P90 (m, 22) | “I still have the handouts/ the ones I got here/ they help me remember.” (P90, m, 22) |
|  | Weekly appointments (2) | P18 (m, 25)  P64 (m, 59) | “When I came here/ I had these weekly appointments/ I always came here to these appointments. They [note: PM+ trainer] talked with me and it helped me/ it helped me a lot/ every week I came here and it was calming me down and after a while I started talking/ I got used to it!” (P64, m, 59) |
|  | Reminder per SMS (2) | P05 (f, 54)  P90 (m, 22) | “I did not miss one appointment. I always came here. I received a call the day before the appointment. She [note: administrative assistant of the project team] reminded me/ she told me that I have an appointment tomorrow/ she reminded me and so I came here.” (P05, f, 54) |
| Learning and practicing | Learning something new (3) | P5 (f, 54)  P64 (m, 59)  P83 (f, 25) | “You have been very friendly and I have learned something new!” (P64, m, 59) |
|  | Further codes: Practicing regularly (1), Practicing breathing technique weekly (1) | | |

^1^n=number of participants whose respond was assigned to a specific code; ^2^f=female, m=male; ^3^I=interviewer; ^4^(...) irrelevant quote
