# Supplementary material for: Self-perceived problems of Afghan asylum seekers and refugees and their experiences with a short psychological intervention
Source: BMC Public Health. 2023 Nov 3;23:2157. doi: 10.1186/s12889-023-17076-7 (PMC10625214; doi:10.1186/s12889-023-17076-7)
Supplement: Supplementary file 3 — Supplementary Material 3 [file 12889_2023_17076_MOESM3_ESM.docx]

**Table S2.**

*Reported effects of PM+ training*

| Theme^1^ | Subcode (n)^2^ | Participant # (gender^3^, age) | Selected quotes (P#, gender^3^, age) |
| --- | --- | --- | --- |
| Mental^a^ | feeling better (10) | P05 (f, 54)  P18 (m, 25)  P25 (m, 36)  P38 (m, 24)  P45 (m, 20)  P47 (f, 36)  P51 (f, 59)  P81 (f, 56)  P90 (m, 22)  P97 (f, 40) | “The training had been good. I joined it and did the movements. Afterwards I felt better. I had a good feeling later.” (P45, m, 20) |
|  | feeling empowered (5) | P01 (f, 50)  P42 (f, 37)  P50 (f, 21)  P66 (f, 35)  P99 (f, 24) | “I used to think that I am worthless. (…) and that I cannot achieve anything. (…) during the training I realized that if I want to do something, I could do it (…) achieve it.” (P50, f, 21) |
|  | being calmer/steadier (5) | P05 (f, 54)  P38 (m, 24)  P42 (f, 37)  P45 (m, 20)  P47 (f, 36) | I^4^: “How did PM+ training affect your everyday life, what changes did participating in PM+ training cause in your life?”  P: “You mean in everyday life?”  I: “Yes”  P: “I have become calmer.” (P42, f, 37) |
|  | being more positive (4) | P01 (f, 50)  P18 (m, 25)  P36 (m, 23)  P66 (f, 35) | “When I wake up in the morning, I think that I'm happy, that I feel good. I try to think positively. And I wish for something good and I think to myself, "in the future everything will be fine again” (P18, m, 25) |
|  | ruminating less (4) | P18 (m, 25)  P36 (m, 23)  P38 (m, 24)  P66 (f, 35) | I: “Have you noticed any changes in your everyday life since taking part in the training?”  P: “Yes of course. I don't have to think so much. And I have to calm down.” (P38, m, 24) |
| Knowledge^b^ | regulating oneself better (11) | P01 (f, 50)  P14 (m, 34)  P18 (m, 25)  P25 (m, 36)  P38 (m, 24)  P42 (f, 37)  P45 (m, 20)  P47 (f, 36)  P83 (f, 25)  P86 (m, 26)  P99 (f, 24) | “Some situations are very stressful. For example, this month I have an appointment for the driver's license. (...). It is difficult because I have to learn a lot before the test and before I go to the test on that day, I will do the breathing exercise that I have learned in training.” (P83, f, 25) |
|  | taking time to solve problems (1) | P36 (m, 23) | “What I learned back then, it was like that/ I thought to myself: "Okay, I started from scratch. Where am I now?" And then I noticed, that life doesn't go all that fast. The problem isn't solved all at once. That is what I have learned.” (P36, m, 23) |
| Interpersonal^c^ | engaging more with people (5) | P31 (m, 21)  P50 (f, 21)  P56 (f, 32)  P81 (f, 56)  P97 (f, 40) | “I wanted to stay alone. Back then, I refrained from my family and other people. But now I like to have better / more contact with my family, with my children, and have more to do with them.” (P97, f, 40) |
|  | Coping better with conflicts/ being more patient (5) | P05 (f, 54)  P18 (m, 25)  P42 (f, 37)  P64 (m, 59)  P97 (f, 40) | I: “What changes has participation in PM + training brought in your everyday life?”  P: “It has already caused changes in how you talk to other people / how you should behave. That was very helpful.”  I: “You noticed that you have treated other people differently since then?”  P: “Yes, exactly, you have to be patient.” (P64, m, 59) |
|  | seeking social support (2) | P31 (m, 21)  P66 (f, 35) | “If I have a problem, I do the breathing exercise or, when I have nightmares and all, I talk to a friend.” (P31, m, 21) |
| Behavioral^d^ | establishing daily structure/activation (5) | P19 (m, 57)  P38 (m,24)  P45 (m, 20)  P51 (f, 59)  P99 (f, 24) | “I used to sleep during the day. I slept a lot. And the psychologist said that it would be better that I did not sleep too much. That I need to get up. That's what I did and I feel better now.” (P51, f, 59) |
|  | being more autonomous (2) | P19 (m, 57), P86 (m, 26) | “I even went shopping, although I haven't done this before. But now I've been doing this since training / after the training and now. That's really helpful.” (P19, m, 57) |
| Physical^e^ | stomach smaller (1) | P25 (m, 36) | “I had a big belly. I breathed a lot, that’s why I have a smaller belly now (...) The guys [persons who are living with the participant] were fun too. They said: “No matter what sadness or depression had been in your stomach, it's all out now. With this breathing training / that's why your stomach got smaller.” (P25, m, 36) |
|  | sleeping better (1) | P05 (f, 54) | “I was able to sleep at night, too.” (P05, f, 54) |
|  | having less headache (1) | P66 (f, 35) | “Usually I always had a headache and I took medication for it. But since I learned these exercises for neck and shoulder area, my headaches have decreased and of course I don't take as many medications as I used to.” (P66, f, 35) |
| insufficient effect | Cannot recognize impact (5) | P14 (m, 34)  P20 (m, 25)  P36 (m, 36)  P50 (f, 21)  P81 (f, 56) | I: “What changes has participating in this program brought for you in everyday life?”  P: ”In everyday life nothing.”  I: “So no change on your life?”  P: “No.“ (P50, f, 21) |
|  | ruminating more (1) | P18 (m, 25) | “I didn't want to talk about the past. Every time I went there [PM + training] and afterwards back home, I spent the whole night thinking about the past. (P18, m, 25) |

Note: ^1^ The categorization was based on Van't Hof , et al. (2018) who found five domains of reported changes after PM+ training; ^2^n=number of participants whose respond was assigned to a specific (sub)code; ^3^f=female, m=male; ^4^I=interviewer
