# Supplementary material for: Self-perceived problems of Afghan asylum seekers and refugees and their experiences with a short psychological intervention
Source: BMC Public Health. 2023 Nov 3;23:2157. doi: 10.1186/s12889-023-17076-7 (PMC10625214; doi:10.1186/s12889-023-17076-7)
Supplement: Supplementary file 4 — Supplementary Material 4 [file 12889_2023_17076_MOESM4_ESM.docx]

| **Table S4**  *Strategies participants continued practicing after completing aPM+* | | |
| --- | --- | --- |
| Strategy (n^1^) | Participants  P# (gender, age^2^) | Selected quotes (P#, gender, age) |
| Managing Stress/ slow breathing (15) | P01 (f, 50)  P05 (f, 54)  P18 (m, 25)  P19 (m, 75)  P25 (m, 36)  P31 (m, 21)  P36 (m, 23)  P42 (f, 37)  P47 (f, 36)  P51 (f, 59)  P64 (m, 59)  P66 (f, 35)  P81 (f, 56)  P83 (f, 25)  P99 (f, 24) | “When I go to bed and the kids are sleeping and I don’t have to do anything else, I do the slow breathing technique/ I concentrate on breathing in and on breathing out/ the one I learned at the training. I practice it for about half an hour before I get to bed. And I do this until today (...). It’s really calming me down. Okay, to be honest, I don’t always do half an hour (laughing)/ at the beginning I did half an hour, but now/ I don’t know/ maybe I don’t have enough time or maybe I don’t take enough time/ I actually don’t know what has happened/ in the last time I just do about 10-15 minutes, but it’s helping me a lot when I experience a lot of stress/ it’s helping me really well!” (P42, f, 37) |
| Strenghtening Social Support (5) | P18 (m, 25)  P31 (m, 21)  P66 (f, 35)  P81 (f, 56)  P97 (f, 40) | “I^3^: Which strategies did you continue to practice after the training?  P: To ask somebody for help. That was really something important for me to learn again.” (P97, f, 40) |
| Get Going, Keep Doing (4) | P1 (f, 50)  P38 (m, 24)  P64 (m, 59)  P66 (f, 35) | “I go out/ that’s what I’ve learned/ that I need to go out and get some fresh air, if it’s possible. Yes, that’s what I’ve learned. I go out, I go for a walk to the park/ I’ve been doing that a lot lately.” (P64, m, 59) |
| Managing Problems (4) | P18 (m, 25)  P47 (f, 36)  P83 (f, 25)  P99 (f, 24) | “I do everything, but especially the problem management/ to solve a problem step by step/ that you can solve a problem with small steps.” (P99, f, 24) |
| Taking a break (1) | P38 (m, 34) | “One was this about studying/ how it is easier to study/ And that I have to take a break between study sessions/ I should take a break and drink coffee, for example.” (P38, m, 34) |
| Looking at the „tree of resources“ (1) | P99 (f, 24) | “It reminds me of the things I achieved so far, my successes…it helps me to think about it and to say: „Okay, I really achieved this and that.” (P99, f, 24) |

*Note.* From 24 participants, 15 practiced slow breathing after the training has ended. The remaining strategies were continued by only a few individuals.

^1^number assigned to the participants within the main study; ²f=female, m=male; ^3^I=Interviewer.
