# Supplementary material for: Self-perceived problems of Afghan asylum seekers and refugees and their experiences with a short psychological intervention
Source: BMC Public Health. 2023 Nov 3;23:2157. doi: 10.1186/s12889-023-17076-7 (PMC10625214; doi:10.1186/s12889-023-17076-7)
Supplement: Supplementary file 5 — Supplementary Material 5 [file 12889_2023_17076_MOESM5_ESM.docx]

| **Table S5**  *Challenges with aPM+* | | | |
| --- | --- | --- | --- |
| Themes | Codes (n^1^) | Participants  P# (gender, age^2^) | Selected quotes (P#, gender, age) |
| Specific aPM+ strategies and interventions | Cannot recall all strategies (7) | P01 (f, 50)  P05 (f, 54)  P31 (m, 21)  P47 (f, 36)  P56 (f, 32)  P81 (f, 56)  P90 (m, 22) | “The strategies that were helpful are in my thoughts/ in my memory. I cannot recall those, which were not helpful.  I^3^: You remember only the strategies that were helpful?  P: Yes. The ones that helped me in my everyday life/ the ones that changed something, I can recall. However, the ones that did not help me/ I cannot see/ I have forgotten them. I don’t use them in my everyday life.” (P81, f, 56) |
|  | Strengthening social support (3) | P25 (m, 36)  P56 (f, 32)  P99 (f, 24) | “Breathing in, breathing out/ I can do that! However, I cannot go to a party or have contact with other people, I cannot do that/ it’s not possible/ I don’t have the opportunity.” (P25, m, 36) |
|  | Managing problems (2) | P83 (f, 25)  P50 (f, 21) | „Yes, one is difficult/ the steps of managing problems/ that is quite difficult/ more difficult than other strategies (laughing).” (P83, f, 25) |
|  | Further codes: Slow breathing (1), Understanding emotions (1), Dealing with feelings of aggression (1), Weekly calendar (1),  Inactivity cycle (1) | | |
| Transfer to everyday life | Didn’t continue after training has ended (3) | P5 (f, 54)  P14 (m, 34)  P36 (m, 23) | “During the training, everything was helpful/ it was good. Then, new problems occurred and I stopped doing anything/ I just stopped.” (P36, m, 23) |
|  | Further codes: Was only helpful during training (1), Didn’t help in everyday life (1) | | |
| Other expectations | Other strategies needed (e.g. dealing with anxiety) (1) | P86 (m, 26) | “Nothing helped against my anxiety/ I suffer from anxiety/ I was afraid, but it (note: aPM+ training) was not helpful.  I: It didn’t help to better deal with your anxiety?  P: No.” (P86, m, 26) |
|  | Further codes: Too less appointments (1), Wanted to “empty my head” (1), Needed concrete help (1) | | |
| Others | Too many questions (about past) (3) | P14 (m, 34)  P18 (m, 25)  P47 (f, 36) | “I didn’t find a solution. Only questions and no answers. It was boring for me to get so many questions and to speak only about one theme every week. I don’t like it when my situation is not recognized/ when my story isn’t acknowledged. That’s not good for me.” (P14, m, 34) |
|  | Unsolvable problems (3) | P50 (f, 21)  P36 (m, 23)  P20 (m, 25) | “I: You said before that the training did not help you. Could you explain your experiences more detailed? I would like to learn how we could improve helping you and others with similar experiences.  P: It depends on the kind of problem one has.  I: It depends on the type of problem?  B: Yes, and I think it does not work for my problem.  I: Which problem did you try to solve?  P: I don’t remember now/ but in general, it does not work for my current problems.”  (P50, f, 21) |
|  | Further codes: Lack of empathy (1), Felt uncomfortable thinking about past (1) | | |

^1^n=number of participants whose respond was assigned to a specific code; ^2^f=female, m=male; ^3^I=interviewer.
