# Supplementary material for: Self-perceived problems of Afghan asylum seekers and refugees and their experiences with a short psychological intervention
Source: BMC Public Health. 2023 Nov 3;23:2157. doi: 10.1186/s12889-023-17076-7 (PMC10625214; doi:10.1186/s12889-023-17076-7)
Supplement: Supplementary file 6 — Supplementary Material 6 [file 12889_2023_17076_MOESM6_ESM.docx]

**Table S6.**

*Suggestions for improving aPM+*

| Codes (n^1^) | Participant # (gender^2^, age) | Selected quotes (P#, gender, age) |
| --- | --- | --- |
| More treatment-sessions (4) | P18 (m, 25)  P47 (f, 36)  P51 (f, 59)  P81 (f, 59) | “In my opinion, although I know that you know much more than I do, because you are a psychologist, psychotherapist, but I think when people get more time, for example, for therapy, it's much better. I also think that it’s important that you get more appointments / treatment faster. Because I can remember when I had been in this situation, it was so difficult that one hour felt like one day. Like 24 hours.” (P18, m, 25) |
| More hands-on practice (2) | P81 (f, 59)  P64 (m, 56) | “I think it would be better if there were more hands-on practice. (…) Some people don't want to go out from home, from their own apartment, and feel inactive. They are always at home. That’s why I think it would be necessary to do more exercises. (…) You had the strategy “problem management” that helps you to get out of a problem when you look closely at it. I think for these people [future Pm+ participants] you really need that kind of hands-on practice. That you do it together with them. Then they can get out of such problems. Solve their own problems.” (P81, f, 56) |
| More information / psychoeducation (1) | P56 (f, 32) | “If you [research team] could give more information, it would be better. For example / life / parenting / work / learning (…) That participants learn to control their nerves (…) and to gain confidence. Because if someone has self-confidence and is independent, other people cannot influence him/her.” (P56, f, 32) |
| Additional drug treatment / further treatment (1) | P47 (f, 36) | “I think it is better for people who have mental health problems to get some medication. Or they should be sent to another psychologist instead of this training. (…) because this training includes only six appointments and six appointments are not enough for people who have difficult psychological problems. It would be better if they go to another psychologist to receive further treatment.” (P47, f, 36) |
| Promote treatment among Afghan men (1) | P42 (f, 37) | “I think that if men also participate, that would be good, because mostly women come to these outpatient clinics. (…) I keep making this proposal to Afghan men, but unfortunately, very few accept it. I also sent seven or eight other women to XX [address where the study took place], but I haven't been able to win the Afghan men over to tell them that they have to go there too. Although I think that, the Afghan men should definitely go there and learn these strategies.” (P42, f, 37) |
| Talk less about the past (1) | P18 (m, 25) | “In my opinion, it is better if the past is only talked about and asked once. Everything important should be asked at the beginning. It should be avoided that you ask the same questions again at other appointments.” (P18, m, 25) |
| Further Codes: Involve friends more actively / encourage participants to recommend the training (1), cancel one strategy (1) | | |

Note: ^1^n=number of participants whose respond was assigned to a specific code; ^2^f=female, m=male
