# Supplementary material for: Self-perceived problems of Afghan asylum seekers and refugees and their experiences with a short psychological intervention
Source: BMC Public Health. 2023 Nov 3;23:2157. doi: 10.1186/s12889-023-17076-7 (PMC10625214; doi:10.1186/s12889-023-17076-7)
Supplement: Supplementary file 7 — Supplementary Material 7 [file 12889_2023_17076_MOESM7_ESM.docx]

**Table S3**

*Recommendation of PM+ training and its strategies*

| Codes (n^1^) | Subcodes (n^1^) | Participant # (gender^2^, age) | Selected quotes (P#, gender^2^, age) |
| --- | --- | --- | --- |
| PM+ was recommended (12) |  | P05 (f, 54)  P18 (m, 25)  P19 (m, 57)  P25 (m, 36)  P36 (m, 23)  P42 (f, 37)  P47 (f, 36)  P64 (m, 59)  P81 (f, 56)  P83 (f, 25)  P86 (m, 26)  P90 (m, 22) | “I have told my children that I am here for this training. Then I also contacted other people (...) I contacted my friends again. And they said, "What happened? We see you again. We hear you again." I told them that I was going to the University of Vienna for this training and they were happy. My children, too, my friends, too. And there was also a mother and daughter, I gave them the address and I said, "You could go there too, if you want." I think the two of them came, too. (…) I was really excited and I was happy about the training and I told everyone about it.” (P81, w, 56) |
| Specific strategy that was passed on (8) | Managing stress (breathing) (6) | P05 (f, 54)  P19 (m, 57)  P25 (m, 36)  P42 (f, 37)  P47 (f, 36)  P83 (f, 25) | “I taught my friend the breathing exercises that I do myself.” (P47, w, 36) |
|  | Self-efficacy (ressource tree) (2) | P36 (m, 23)  P42 (f, 37) | P: “I also drew the tree.”  I^3^: “Aha, did you draw the tree [strategy 5: tree of ressources] with them?  P “Yes, I did. I started it with them. I told them how to start life.” (P36, m, 23) |
|  | Managing problems (1) | P83 (f, 25) | “The breathing exercise and the steps of managing problems, too.” (P83, w, 25) |
|  | Inactivity (1) | P19 (m, 57) | “I have a relative here, who has had such problems as I did and I told him: "Please, you go out, you go for a walk and do not stay at home.”” (P19, m, 57) |
|  | No precise strategy given (1) | P18 (m, 25) | “Yes, many of my friends. Those I know who have problems like me. I told them that I was there [at PM + training]. I showed them how to do it and then they did it, too. And it worked for them, too.” (P18, m, 25) |

^1^n=number of participants whose respond was assigned to a specific (sub)code; ^2^f=female, m=male; ^3^I=interviewer
